# The Influence of Protective Headgear on the Visual Field of Recreational-Level Skiers

**DOI:** 10.3390/ijerph191710626

**Published:** 2022-08-25

**Authors:** Mateja Očić, Ivan Bon, Lana Ružić, Vjekoslav Cigrovski, Tomislav Rupčić

**Affiliations:** Laboratory for Sports Games, Faculty of Kinesiology, University of Zagreb, 10000 Zagreb, Croatia

**Keywords:** ski helmet, ski goggles, peripheral vision, vision screening, head injuries, preventive measures, risk factors

## Abstract

The benefit of protective headgear for recreational skiers is an ongoing debate in the snow sports industry, and there are a lot of opposing opinions. Due to the dynamic conditions in which winter sports are performed, athletes demand rapid and constant processing of visual information. A sufficient level of anticipation helps athletes to properly position themselves to reduce the forces transferred to the head or even move to avoid a collision. To objectively identify the impact of protective headgear on the visual field when skiing, it is necessary to conduct suitable measurements. The sample consisted of 43 recreational-level skiers (27 M, 16 F; age 31.6 ± 8.23 years). A predefined testing protocol on an ortoreter was used to assess the visual field for three conditions of wearing protective headgear. Differences in perceived visual stimuli between the three conditions were evaluated by repeated measures analysis of variance (ANOVA). Based on the observed results, it can be concluded that the combination of wearing a ski helmet and ski goggles significantly negatively influences visual performance in a way that the visual field is narrowed, for both helmet users and non-users, only when comparing the tested conditions. When comparing helmet users and non-users, there are no differences in the amount of visual impairment; therefore, the habit of wearing a helmet does not influence the ability of perceiving visual stimuli.

## 1. Introduction

The popularity of winter sports is constantly growing, as well as the trend of high speed and an adrenaline rush on the ski slopes. Consequently, ski slopes have become more crowded, and the risk of collisions and accidents has grown significantly, as well as the risk and number of injuries. Thus, the skier is more exposed in these conditions, and this could lead to an increased risk of injuries on the ski slopes. Moreover, the popularity of freestyle riding, off-piste skiing, and snow park use is also growing, which could affect the increased number of injuries. One of the most serious injuries are of the head, which account for 9% to 20% of overall reported skiing and snowboarding injuries. Furthermore, a head injury is also the leading cause of a fatal injury among skiers and snowboarders. Therefore, in order to minimize the risk of severe head injuries, ski helmets are widely used [1,2,3,4,5].

In competitive skiing, ski helmets are mandatory for skiers in all of the official FIS (International Ski and Snowboard Federation) competitions. However, ski resorts do not typically require helmet use for adult recreational skiers. Even though they are not mandatory, helmet use rate among recreational skiers has increased over the last decade from 16% to 25% in 2002/2003, up to 73–89% in 2013/2014 [6,7].

The benefit of safety headgear is certainly an ongoing debate in the snow sports industry for recreational-level skiers, and there are a lot of opposing opinions regarding the topic. For example, some propose that ski helmets encourage increased levels of risk taking. The false sense of security in helmet users might cause them to engage in more dangerous behavior that exceeds their normal level of acceptable risk [8,9,10,11,12].

Moreover, common arguments for helmet non-use are impaired hearing and impaired vision [13,14,15,16]. In addition, it has been suggested that a helmet might alter skiers’ ability to sense the surroundings, potentially leading to dangerous situations.

When discussing visual abilities, for sports in general and for overall athletic performance, vision is certainly an important factor. It enables the advanced ability to identify and react to peripheral stimuli, quickly shift gaze, and track objects while in motion. Enhanced visual reaction time, visual memory, visual acuity, and visual discrimination have been shown to have a direct impact on improved sport performance and reduced locomotor injury risk. Peripheral vision is very important for performing a wide range of daily activities [17,18], but its importance is even more highlighted in sports due to perception of the visual stimuli located outside the focus of primary area.

Due to the specific and dynamic conditions in which winter sports are performed, they demand rapid and constant processing of visual information. Based on visual feedback, skiers must make quick judgements and adapt their actions, such as speed, direction, or body position [19,20].

Heightened peripheral vision is a key to skiers’ ability to anticipate a collision on the ski slopes. Nowadays it is even more important due to crowded slope conditions. A sufficient level of anticipation helps athletes to properly position themselves, to reduce the forces transferred to the head during an impact or even move to avoid a collision. In this way, skiers can reduce injury risk. When a skier does not anticipate a collision, the head tends to be the first point of contact, and the resulting head impact forces are more severe [21,22].

Although the importance of peripheral vision in alpine skiing is clear, there are a limited number of studies focused on visual perception. Especially, there are not many studies focused on peripheral vision and the influence of protective headgear on the visual abilities of recreational skiers. The main reason might be due to technical limitations and the complexity of the testing procedures necessary to access visual abilities.

Modern technology designed for visual assessment can also be used for testing procedures in conditions where a subject wears various protective headgear (helmet, goggles, sunglasses). To gain valid data and objectively identify the impact of a ski helmet, ski goggles, and sunglasses on the visual field when skiing, it is necessary to conduct suitable measurement, first in laboratory conditions and then in specific conditions on the ski slopes.

Due to mentioned, the aim of this research is to identify whether a ski helmet, ski goggles, and sunglasses in three tested combinations affect skiers’ visual field, when tested in laboratory conditions. Moreover, the aim is to compare the results for helmet users and non-users.

We hypothesize that in the laboratory-testing procedure, the impairment of the visual field is going to be the highest when wearing a ski helmet and ski goggles combined, for both prior helmet users and non-users. However, we assume there would be differences in the results for helmet users and non-users, i.e., that the visual impairment would be higher for helmet non-users when combining a ski helmet and ski goggles.

## 2. Materials and Methods

### 2.1. Participants

The sample consisted of 43 recreational-level skiers (27 M, 16 F; age 31.6 ± 8.23 years). Participants did not report any prior injuries that could affect their skiing technique. They were asked about any prior serious medical conditions regarding the vision system (all declined) and if they had worn a ski helmet previously (helmet users *n* = 22; helmet non-users *n* = 21). Since they served as their own controls, it was not extremely important to evaluate their exact vision status. All participants were recreational-level alpine skiers who had finished basic alpine skiing school and have been skiing 6–10 years continuously (7 to 10 days each winter season). Moreover, all of the helmet users reported that they have been using the helmet continuously since their first skiing day. They gave written consent to participate in this study after being informed in detail about aims and protocol of the investigation. Ethics Committee of the Faculty of Kinesiology, University of Zagreb (Croatia) approved the study, which was performed following the ethical standards of the Declaration of Helsinki.

### 2.2. Variables and Equipment

The overall number of perceived visual stimuli was observed (Overall_n). Moreover, results were obtained separately for 5 regions of ortoreter (vision-screening instrument). The regions were: upper-left region—Upper_L, upper-right region—Upper_R, upper-middle region—Upper_M, middle region—Middle, and down region—Down. Each of the predefined conditions were tested (1—wearing only ski cap (control condition used as a baseline), 2—wearing ski cap and sunglasses, 3—wearing ski goggles and ski helmet).

For testing procedure in laboratory conditions, Optovist EU ortoreter (Vistec, Jena, Germany) was used to assess the visual field. Due to its construction, mentioned vision-screening instrument enables assessing visual field when wearing not only the cap but also sunglasses or ski goggles and ski helmet combined. The test, which was used for the purposes of this investigation, is related to perception of 28 LED visual stimuli integrated in the instrument. LED stimuli were later divided into 5 different regions for the purposes of analysing visual field. This instrument is commonly used for detecting various aspects of vision as well as peripheral vision. The reliability and validity of using Optovist screening instrument for analysing various aspects of vision were confirmed in previous studies of similar activities [23,24,25,26].

### 2.3. Protocol of Investigation

All the included participants followed the same protocol. Firstly, the appropriate size of ski helmet, ski goggles, and sunglasses were individually chosen to suit each participant. A conventional ski helmet (Briko, approved: CE EN1077) was used as well as a standard ski cap (50% virgin wool, 50% acrylic), standard ski goggles (Model: Briko Homer P1), and standard sunglasses (Model: Bliz Hybrid). The ski helmet was available in 4 different sizes (small, medium, large, extra-large) to ensure an optimal fit of the helmet for each tested participant. During the measurements, the ski cap had to be worn so that both ears were fully covered.

**Figure 1:**
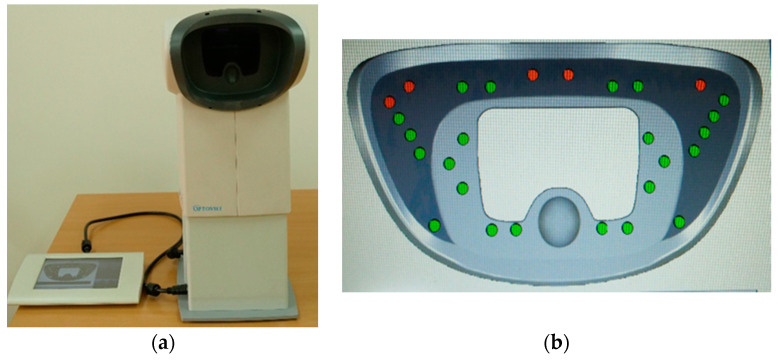
(**a**) Vision-screening instrument (ortoreter); (**b**) test preview on ortoreter (red dots—unregistered visual stimuli by participant; green dots—registered visual stimuli by participant).

**Figure 2:**
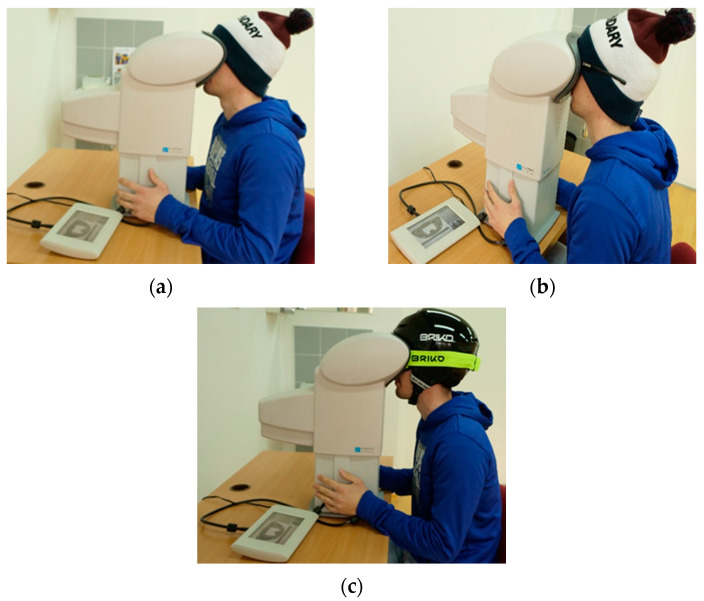
(**a**) Testing protocol using only ski cap; (**b**) testing protocol using ski cap and sunglasses; (**c**) testing protocol using ski helmet and ski goggles.

For assessing visual field, participants underwent predefined test, which was conducted in a room without any background noises or bright lights that could affect the results. The aim was to signal with a simple yes/no answer when LED light flashes in different regions of the examined field. Overall, 28 LED lights flashed randomly in defined regions during testing procedure, so a learning effect was not possible (Figure 1). Prior to the actual visual stimuli localization tests, all participants underwent a short trial phase to familiarize themselves with the test setup. Afterwards, the testing procedure was repeated 3 times, each time testing different conditions—first time wearing only a ski cap, second time with ski cap and sunglasses, and third time wearing ski helmet and ski goggles combined (Figure 2). Measuring device was used in accordance with the instructions of the manufacturer, and the test setup was adapted taking into consideration the different size of ski helmet suiting each participant. In later analysis, overall result and result divided in regions were analysed for each condition.

### 2.4. Statistical Analysis

With the use of the G*power program 3.1.9.7 (University od Dusseldorf, Dusseldorf, Germany), the sample size was calculated (*n* = 40) that was needed for testing procedure with statistical significance of *p* < 0.05; statistical power 0.80; effect size 0.30; and 2 groups. Statistical package Statistica version 13.5.0.17 (TIBCO Software Inc., Palo Alto, CA, USA) was used for data analysis. Basic descriptive parameters for all measured variables were calculated. The normality of data distribution was tested by the Shapiro–Wilk test. Differences in perceived visual stimuli between the 3 conditions were evaluated by repeated measures analysis of variance (ANOVA) and Tukey’s post hoc test for determining the differences between tested conditions. The results were considered significant when *p* < 0.05.

## 3. Results

When conducting ANOVA for repeated measures for helmet users, there were statistically significant differences between the observed conditions in the visual field measured on the ortoreter instrument (F = 26.74; *p* = 0.00). The basic descriptive parameters of each region of visual field measured for examinees who had used a helmet before, along with Tukey’s post hoc results, are presented in Table 1.

Results of the ANOVA repeated measures model determined significant differences among the helmet users in the overall number of perceived visual stimuli and in four out of the five regions of the visual field (*p* < 0.01). The only region that did not differ between three measured conditions was the Middle region. Moreover, in overall numbers of perceived visual stimuli, there was a significant difference between tested conditions (*p* < 0.01).

When observing results in Table 1, regarding the Upper_L region, Tukey’s post hoc shows significant difference between conditions 1–3, *p* < 0.01, and conditions 2–3, *p* < 0.01. In the Upper_R region, similar differences were found to be significant (conditions 1–3, *p* < 0.01; conditions 2–3, *p* < 0.01). In the Upper_M region, significant differences were found between conditions 1–3, *p* < 0.01, and conditions 2–3, *p* < 0.01. In the Middle region, there were no significant differences found between conditions. Regarding the Down region, a significant difference was determined between conditions 2–3, *p* = 0.01. When observing the overall number of noticed LED stimuli, there is a significant difference between conditions 1–3, *p* < 0.01, and conditions 2–3, *p* < 0.01.

When conducting ANOVA for repeated measures for helmet non-users, there were statistically significant differences between the observed conditions (F = 18.98; *p* = 0.00). Basic descriptive parameters of each region of visual field measured for examinees who never used a helmet before, along with Tukey’s post hoc results, are presented in Table 2.

Results of the ANOVA repeated measures model determined differences in the overall number of perceived visual stimuli and in four out of the five observed regions (*p* < 0.01) for helmet non-users. The only visual field region that did not differ between the three conditions was the Middle region. Moreover, in the overall numbers of perceived visual stimuli, there was a significant difference between the tested conditions (*p* < 0.01).

Furthermore, the Tukey’s post hoc results presented in Table 2 showed significant differences in perceiving the visual stimuli on the ortoreter instrument between three conditions. Regarding the Upper_L region, Tukey’s post hoc showed significant difference between conditions 1–3, *p* < 0.01, and conditions 2–3, *p* < 0.01. In the Upper_R region, similar differences were found to be significant (conditions 1–3, *p* < 0.01, conditions 2–3, *p* < 0.01). In the Upper_M region, significant differences were found between conditions 1–3, *p* < 0.01, and conditions 2–3, *p* < 0.01. In the Middle region, there were no significant differences found between conditions. Regarding the Down region, a significant difference was determined between conditions 2–3, *p* = 0.04. When observing the overall number of LED stimuli, a significant difference was found between conditions 1–3, *p* < 0.01, and conditions 2–3, *p* < 0.01.

## 4. Discussion

The obtained results confirm the highest visual field impairment occurs when wearing ski helmet and ski goggles combined, for both helmet users and non-users. When observing the five regions of the visual field separately, significant differences were found between the tested conditions in four out of the five regions. The only region that did not differ between the tested conditions was the Middle region. Our results do not confirm the hypothesis regarding the differences in gained results for helmet users and non-users when observing the role of ski helmet and ski goggles on visual impairment. The results indicate that there are no differences in results when comparing helmet users and non-users, even though we expected visual impairments to be higher for helmet non-users.

When comparing the results between cap-only use (condition 1) and a cap combined with sunglasses (condition 2), there were no statistically significant differences between the tested regions for both helmet users and non-users. On the other hand, both conditions 1 and 2 differed from a helmet combined with goggles (condition 3) between users (four regions) and non-users (three regions).

A similar study regarding the impact of helmet and goggles on visual field was conducted by Ruedl et al. [19], who investigated the differences in reaction time to peripheral stimuli when testing the use of ski equipment in various conditions. They conducted a compensatory tracking task (CTT), where the lowest mean reaction time was measured for cap-only use (477.3 ± 16.6), which was not different from helmet-only use (478.5 ± 9.1, *p* = 0.911). However, reaction time was significantly longer for cap-and-goggles use (514.1 ± 20.8, *p* = 0.005) and for helmet-and-goggles use (497.6 ± 17.3, *p* = 0.017), when compared to cap-only use. They concluded that wearing only a ski helmet did not impair peripheral reaction time during a CTT, but the addition of ski goggles to a helmet negatively impacted peripheral-vision reaction time. Those results are in accordance with our study, when observing the influence of helmet and goggles on visual impairment, but our testing focus was not on the differences in reaction time but on defining the regions of the visual field that were affected the most.

In our study, when observing the condition that includes wearing a helmet and goggles, for both helmet users and non-users, higher visual impairment compared to other regions was found in the three upper regions, and the highest was found in the upper-middle region (users—0.45 ± 0.83, *p* = 0.00; non-users—0.79 ± 0.97, *p* = 0.00). Similar comparison studies from various sports and activities in which protective headgear use is common can also be used as a reference for alpine skiing.

For example, similar results regarding the observed regions when testing the visual field were found in a study conducted by Wilkins and coauthors [27]. The authors tested different visual field regions when wearing cricket helmets. The tested regions were upper-central visual field, central visual field, lower-central visual field, and inferior visual field. An analysis of the locations in which the visual field was restricted revealed similar to our study, that most changes occurred in the superior visual field.

It is expected that the mentioned region has the lowest score, since it is not in the main focus of the eye. The only problem in our study is that it is not clear whether a helmet or goggles affect visual impairment. However, when we compare our findings with some other studies, it can be concluded that the influence on the visual field is the highest when combining the helmet with some other headgear, such as goggles. Thus, in order to make valid conclusions, it is necessary to provide testing protocols consisting of conditions that include wearing a helmet and goggles separately as well as together.

Kauffman et al. [28] reported that female athletes wearing sports goggles had impaired detection of peripheral visual stimuli, which supports the statement of the negative effect of goggles on the visual field and reactions. Furthermore, when observing separately the role of ski goggles on the visual field, Ruedl et al. [29] concluded that peripheral vision seems to be limited due to the construction of the ski goggles, not due to the ski helmet. In this study, the mean reaction time of helmet and goggles use together was on average about 30–35 ms longer than of helmet use alone.

Moreover, Ing et al. [30] conducted research on hockey, and their findings indicate that a hockey visor and sports goggles adversely affect peripheral vision, especially in the far temporal field, which is greater than 60° from fixation.

One of the reasons for impaired peripheral vision when wearing goggles could be protection design as well as the distance between the protective material and the eye. Ski and sports goggles are characterized by relatively thick frames that are fixed very close to the orbital region of the face, which may cause greater obstruction to peripheral vision.

There are several studies focusing exclusively on the role of a helmet on visual field impairment. For example, in a study conducted by Kramer et al. [22], the main focus was on the effect of protective helmets from various sports on vision and sensory performance. Their conclusions were connected to a decreased ability to react to stimuli that were located in the corners of the Senaptec screen, i.e., the response was slower to visual targets in periphery when wearing a helmet. The response time is crucial in sports, because the additional time it takes an individual to respond to a target when wearing a helmet translates to a slower response to an incoming threat. In the case of skiing, a slower reaction and narrowed visual field increase the risk of collision with other skiers on the ski slopes.

Similar findings regarding the impact of helmet on visual field were also confirmed in American football. Miller et al. [31] investigated the effect of headgear on peripheral-vision reaction time and visual target detection in American football players. Their findings reveal that wearing a helmet or a helmet with an eye shield decreased the total hit score during peripheral-reaction-time tests. Those conclusions can be translated to the results of our study because they confirm that a helmet in combination with goggles, which are used as an eye shield, influence the visual ability of perceiving peripheral stimuli. In both cases, the peripheral visual field is narrowed due to eye shield construction.

These findings may provide important information for players and coaches from different sport activities, especially dynamic ones such as skiing, concerning how protective headgear affects visuomotor ability.

### Limitations

In our study, a helmet and goggles were tested only combined, therefore, it cannot provide insight into how the visual field would be impaired in case of wearing a helmet and goggles separately. Thus, the main limitation of our study is the fact that we did not test all the possible combinations of wearing protective headgear but rather concentrated on the most commonly used ones in practice. In future studies, the same testing protocol with all of the possible combinations of wearing a ski cap, a helmet, sunglasses, and goggles would be needed, enabling a clearer insight into the effect of each headgear separately and in various combinations.

Moreover, the testing protocols on the ski slope should be considered to mimic the specific conditions of skiing on the ski slopes. In that way, valid conclusions of the influence of protective headgear in skiing on the visual field and reaction time to peripheral stimuli could be gained. That information could be crucial for understanding the potential detrimental effects of headgear to visual performance, which could possibly lead to better anticipation and faster response to visual stimuli. Consequently, the safety aspects of skiing would be significantly improved, as the injury risks decrease and performance improves.

Age group certainly plays a significant role in the gathered results. Different results could potentially be obtained if the participants were older, due to the greater possibility of visual impairments in that age group. In future studies, it would be interesting to incorporate and compare different age groups to get a clear insight into the possible differences of the gained results.

## 5. Conclusions

Based on the observed results, it can be concluded that a combination of wearing a ski helmet and ski goggles significantly negatively influences visual performance, in the way that the visual field is narrowed, for both helmet users and non-users, only when comparing tested conditions. The peripheral vision is narrowed, which is especially emphasized in perceiving visual stimuli in upper regions, followed by lower regions of the visual field. When comparing the results of helmet users and non-users, there are no differences in the amount of visual impairment, i.e., the habit of wearing a helmet does not influence the ability of perceiving visual stimuli.

In order to mimic alpine skiing conditions as much as possible, it would be necessary to conduct similar research, while incorporating all of the possible combinations of wearing protective headgear. It would enable valid conclusions about the influence of each piece of headgear separately and in combination on peripheral vision and the perception of visual stimuli. Moreover, to make general conclusions about the influence of protective headgear on visual performance, tests of the protocols on the ski slopes should be designed and conducted.

The gained information could help in improving the safety aspects of skiing and the overall performance of skiers, with the highlighted importance of understanding how protective headgear influences the visual abilities of recreational-level skiers.

## Figures and Tables

**Table 1:** Basic descriptive statistical parameters and Tukey’s post hoc results of ANOVA for each region for helmet users (*n* = 22) (1—wearing only ski cap, 2—wearing ski cap and sunglasses, 3—wearing ski helmet and ski goggles).

Variable	1 Mean ± SD	2 Mean ± SD	3 Mean ± SD	1 vs. 2 (*p*)	1 vs. 3 (*p*)	2 vs. 3 (*p*)
**Upper_L**	3.66 ± 1.01	3.24 ± 1.24	1.14 ± 0.74	0.28	0.00 *	0.00 *
**Upper_R**	3.76 ± 1.15	3.31 ± 0.97	1.07 ± 0.75	0.19	0.00 *	0.00 *
**Upper_M**	5.55 ± 0.78	4.97 ± 1.27	0.45 ± 0.83	0.07	0.00 *	0.00 *
**Middle**	6.00 ± 0.00	6.00 ± 0.00	5.97 ± 0.19	1.00	0.44	0.44
**Down**	5.79 ± 0.41	5.93 ± 0.37	5.48 ± 0.74	0.59	0.07	0.01 *
**Overall**	24.76 ± 2.56	23.45 ± 3.21	14.10 ± 2.21	0.16	0.00 *	0.00 *

Legend: * *p* < 0.05; Upper_L—upper-left region LED stimuli in instrument; Upper_R—upper-right region LED stimuli in instrument; Upper_M—upper-middle region LED stimuli in instrument; Middle—middle region LED stimuli in instrument; Down—down region LED stimuli in instrument; Overall—overall number of LED stimuli.

**Table 2:** Basic descriptive statistical parameters and Tukey’s post hoc results of ANOVA for each region for helmet non-users (*n* = 21) (1—wearing only ski cap, 2—wearing ski cap and sunglasses, 3—wearing ski helmet and ski goggles).

Variable	1 Mean ± SD	2 Mean ± SD	3 Mean ± SD	1 vs. 2 (*p*)	1 vs. 3 (*p*)	2 vs. 3 (*p*)
**Upper_L**	4.00 ± 0.96	3.92 ± 0.92	1.00 ± 0.39	0.97	0.00 *	0.00 *
**Upper_R**	4.36 ± 0.63	3.85 ± 0.95	1.36 ± 0.63	0.20	0.00 *	0.00 *
**Upper_M**	5.79 ± 0.43	5.07 ± 1.07	0.79 ± 0.97	0.09	0.00 *	0.00 *
**Middle**	6.00 ± 0.00	5.93 ± 0.27	6.00 ± 0.00	0.45	1.00	0.45
**Down**	5.86 ± 0.36	5.93 ± 0.27	5.36 ± 0.93	0.95	0.08	0.04 *
**Overall**	26.00 ± 1.88	24.71 ± 2.05	14.50 ± 1.95	0.21	0.00*	0.00 *

Legend: * *p* < 0.05; Upper_L—upper-left region LED stimuli in instrument; Upper_R—upper-right region LED stimuli in instrument; Upper_M—upper-middle region LED stimuli in instrument; Middle—middle region LED stimuli in instrument; Down—down region LED stimuli in instrument; Overall—overall number of LED stimuli.

## Data Availability

The data presented in this study are available on request from the corresponding author. The data are not publicly available due to its huge size and participants’ privacy protection.

## References

[1] Ackery A., Hagel B.E., Provvidenza C., Tator C.H. (2007). An international review of head and spinal cord injuries in alpine skiing and snowboarding. Inj. Prev..

[2] Jung C.S., Zweckberger K., Schick U., Unterberg A.W. (2011). Helmet use in winter sport activities-attitude and opinion of neurosurgeons and non-traumatic-brain-injury-educated persons. Acta Neurochir..

[3] Steenstrup S.E., Bere T., Bahr R. (2014). Head injuries among FIS World Cup alpine and freestyle skiers and snowboarders: A 7-year cohort study. Br. J. Sports Med..

[4] Stenroos A., Handolin L. (2018). Head injuries in urban environment skiing and snowboarding: A retrospective study on Injury severity and Injury mechanisms Scandinavian. J. Surg..

[5] Owens B.D., Nacca C., Harris A.P., Feller R.J. (2018). Comprehensive review of skiing and snowboarding injuries. J. Am. Acad. Orthop. Surg..

[6] National Ski Areas Association Helmet Usage and Safety Fact Sheet: National Ski Areas Association (NSAA). https://www.nsaa.org/media/209466/HelmetFactSheet_10_1_2014.pdf.

[7] Baschera D., Hasler R.M., Taugwalder D., Exadaktylos A., Raabe A. (2015). Association between head injury and helmet use in alpine skiers: Cohort study from a Swiss level 1 trauma center. J. Neurotrauma.

[8] Hagel B.E., Pless I.B., Goulet C., Platt R.W., Robitaille Y. (2005). Effectiveness of helmets in skiers and snowboarders: Case-control and case crossover study. BMJ.

[9] Scott M.D., Buller D.B., Andersen P.A., Walkosz B.J., Voeks J.H., Dignan M.B., Cutter G.R. (2007). Testing the risk compensation hypothesis for safety helmets in Alpine skiing and snowboarding. Inj. Prev..

[10] Russel K., Christie J., Hagel B.E. (2010). The effects of helmets on the risk of head and neck injuries among skiers and snowboarders: A metaanalysis. CMAJ.

[11] Ruzic L., Tudor A. (2011). Risk-taking behavior in skiing among helmet wearers and nonwearers. Wilderness Environ. Med..

[12] Ruedl G., Abart M., Ledochowski L., Burtscher M., Kopp M. (2012). Self-reported risk taking and risk compensation in skiers and snowboarders are associated with sensation seeking. Accid. Anal. Prev..

[13] Evans B., Gervais J.T., Heard K., Valley M., Lowenstein S.R. (2009). Ski patrollers: Reluctant role models for helmet use. Int. J. Inj. Control Safety Promotion..

[14] Ruedl G., Pocecco E., Sommersacher R., Gatterer H., Kopp M., Nachbauer W., Burtscher M. (2010). Factors associated with self-reported risk taking behaviour on ski slopes. Br. J. Sports Med..

[15] Tudor A., Ruzic L., Bencic I., Sestan B., Bonifacic M. (2010). Ski helmets could attenuate the sounds of danger. Clin. J. Sport Med..

[16] Adhilakshmi A., Karthiga U.K., Ashok N.J. (2016). Auditory and visual reaction time and peripheral field of vision in helmet users. J. Bangladesh Soc. Physiol..

[17] Larson A.M., Loschky L.C. (2009). The contributions of central versus peripheral vision to scene gist recognition. J. Vis..

[18] Strasburger H., Rentschler I., Jüttner M. (2011). Peripheral vision and pattern recognition: A review. J. Vis..

[19] Ruedl G., Herzog S., Schöpf S., Anewanter P., Geiger A., Burtscher M., Kopp M. (2011). Do ski helmets affect reaction time to peripheral stimuli?. Wilderness Environ. Med..

[20] Decroix M., Wazir M.R.W.N., Zeuwts L., Deconinck F.F.J.A., Lenoir M., Vansteenkiste P. (2017). Expert–non-expert differences in visual behaviour during alpine Slalom skiing. Hum. Mov. Sci..

[21] Schläppi O., Urfer J., Kredel R. (2016). Visual perception in alpine ski racing. A qualitative analysis based on interviews with top-level athletes. Sportwiss.

[22] Kramer M.R., Teel E.F., Wasserman E.B., Mihalik J.P. (2021). Effect of Protective Helmets on Vision and Sensory Performance in Healthy Men. Athl. Train. Sports Health Care.

[23] Wilhelm H., Peters T., Durst W., Roelcke S., Quast R., Hütten M., Wilhelm B. (2013). Assessment of mesopic and contrast vision for driving licences: Which cut-off values, which methods are appropriate?. Klin. Mon. Fur Augenheilkd..

[24] Theis S., Alexander T., Wille M. The nexus of human factors in cyber-physical systems: Ergonomics of eyewear for industrial applications. Proceedings of the 2014 ACM International Symposium on Wearable Computers: Adjunct Program (ISWC ‘14 Adjunct), Association for Computing Machinery.

[25] Bergmann L.C., Darius S., Kropf S., Böckelmann I. (2016). Measurement of contrast vision: Mesopic or photopic vision?: Comparison of different methods for measuring contrast sensitivity within the framework of driving licence regulations. Der Ophthalmol. Z. Der Dtsch. Ophthalmol. Ges..

[26] Westhoven M., Paul D., Alexander T. Head turn scaling below the threshold of perception in immersive virtual environments. Proceedings of the 22nd ACM Conference on Virtual Reality Software and Technology—VRST 2016.

[27] Wilkins L., Mann D., Dain S., Hayward T., Allen P. (2019). Out with the old, in with the new: How changes in cricket helmet regulations affect the vision of batters. J. Sports Sci..

[28] Kauffman D.C., Clark J.F., Smith J.C. (2015). The influence of sport goggles on visual target detection in female intercollegiate athletes. J. Sports Sci..

[29] Ruedl G., Pocecco E., Wolf M., Schöpf S., Burtscher M., Kopp M. (2012). Does Listening to Music with an Audio Ski Helmet Impair Reaction Time to Peripheral Stimuli?. Int. J. Sports Med..

[30] Ing E., Ing T., Ing S. (2002). The effect of a hockey visor and sports goggles on visual function. Can. J. Ophthalmol..

[31] Miller R.A., Rogers R.R., Williams T.D., Marshall M.R., Moody J.R., Hensarling R.W., Ballmann C.G. (2019). Effects of Protective American Football Headgear on Peripheral Vision Reaction Time and Visual Target Detection in Division I NCAA Football Players. Sports.

